# Learning by Doing: Chinese Male Nursing Students' Experiences of Developing and Demonstrating Empathy When Caring for Patients in the Oncology Wards

**DOI:** 10.1002/nop2.70481

**Published:** 2026-03-08

**Authors:** Xue Chen, Fang‐Tuan Wu, Li‐Hung Lee, Cheng‐I Yang, Xiao‐Chen Lyu

**Affiliations:** ^1^ Department of Nursing West Anhui Health Vocational College Lu'an Anhui China; ^2^ Department of Geriatrics the First Affiliated Hospital of Anhui Medical College Hefei Anhui China; ^3^ Department of Nursing Hungkuang University Taichung Taiwan; ^4^ Outpatient Operating Room the First Affiliated Hospital of Wannan Medical College Wuhu Anhui China

**Keywords:** China, empathy, male nursing student, oncology, qualitative study

## Abstract

**Aim:**

To explore the experiences of Chinese male nursing students in developing and demonstrating empathy when caring for patients in oncology wards.

**Design:**

A qualitative descriptive study design was employed.

**Methods:**

Data were collected through semi‐structured interviews with 23 male nursing students who had completed theoretical coursework and were undergoing clinical placements in oncology wards in Anhui Province, China. Thematic analysis was used to analyse the transcribed interviews.

**Results:**

Three themes were identified, as follows: (1) challenges in translating empathy into practice, (2) determinants of empathy and (3) approaches to demonstrating empathy. Male nursing students lack systematic learning on how to demonstrate empathy, both in the classroom and during clinical placements. Although they can roughly understand the meaning of empathy from multiple informal sources, they find it difficult to implement it in practice, especially in terms of language expression. In addition, their interactive relationships with patients affect their willingness to show empathy.

**Conclusion:**

Schools and clinical educators should integrate relevant courses and training into the curriculum to enhance nursing students' knowledge and skills in demonstrating empathy. For male nursing students, it is especially important to understand their experiences and needs while also focusing on improving their ability to express empathy through verbal communication.

**Patient or Public Contribution:**

None.

## 1 Introduction

Empathy is the ability to understand and connect with another person's emotions by imagining oneself in the other's situation, often described as putting oneself in another's shoes (Jiao et al. 2022). It involves perceiving, interpreting and effectively conveying an understanding of others' feelings (Babaii et al. 2021). As a fundamental component of nursing care, empathy is deeply integrated into the caring process. It enables nurses to comprehend patients' emotions, experiences and psychosocial needs, facilitating compassionate care and a holistic understanding of the patient's perspective (Wu 2021). Moreover, empathy plays a pivotal role in shaping the quality of patient care and strengthening the therapeutic relationship between nurses and patients (Babaii et al. 2021). Research on nurse–patient communication underscores the significance of empathy in delivering high‐quality care. As a core element of nurse–patient interactions, it serves as a vital clinical indicator of effective nursing practice (Wu 2021).

Literature emphasises the importance of education in fostering empathy within the modern nursing environment, and nursing educators play a crucial role in enhancing nurses' empathy skills (Heggestad et al. 2018). Multiple studies have explored this topic, yielding diverse findings, yet they consistently highlight that empathy is a skill that can be cultivated and developed (Cunico et al. 2012). Clinical placements serve as key opportunities for nursing students to learn and demonstrate empathy because exposure to the clinical setting and interactions with patients can help cultivate nursing students' empathy and shape their professional identity (Wang et al. 2022). Additionally, patients with cancer often face intense emotions, including fear, anxiety, anger and sadness, making empathetic care essential for their overall well‐being. Empathy is widely recognised as a crucial component of oncology care, particularly in challenging environments such as cancer wards, where patients experience significant physical and emotional distress. Research suggests that higher patient‐perceived empathy is associated with greater satisfaction, improved self‐efficacy and reduced emotional distress following medical consultations (Sanders et al. 2021).

Furthermore, existing literature suggests that men and women exhibit distinct empathy traits, which may also influence how they learn and develop empathy (Cunico et al. 2012). It has been argued that, as compared with those of male nurses, the empathic skills of female nurses and their empathic tendencies tend to be higher (Alkan 2017). Research also indicates that female nursing students tend to demonstrate greater emotional expressiveness, which enhances their ability to empathise. As a result, they often exhibit higher levels of empathy in clinical patient care than do their male counterparts (Jiao et al. 2022). Additionally, a higher number of female participants attend training sessions, suggesting potential differences in engagement with empathy‐focused education (Cunico et al. 2012).

Despite the increasing presence of male nursing students in the profession, research on how these students develop and express empathy in clinical settings remains limited. Most existing studies have focused on general nursing populations or female nursing students (Alkan 2017), leaving a gap in understanding the unique experiences and challenges that male nursing students face in cultivating empathy. Further research is needed to explore factors influencing the development of empathy in male nursing students, including educational approaches, clinical experiences and societal expectations regarding emotional expression in healthcare settings.

Building on this gap, existing studies on empathy in nursing education have often treated nursing students as a homogeneous group, with limited attention paid to potential gender differences. In the Chinese context, such gender differences may be particularly salient, as expressions of empathy are shaped not only by professional training but also by societal expectations and culturally embedded gender roles. Influenced by Confucian values and norms associated with hegemonic masculinity, Chinese men are often socialised to emphasise emotional restraint, strength and self‐control (Low 2012), which may affect how empathy is experienced and demonstrated in caregiving roles. Research on empathy development suggests that such gendered socialisation processes can shape how individuals learn to express care and emotional responsiveness in professional contexts (Cassels et al. 2010).

To address this gap, this study employed a qualitative research approach to explore Chinese male nursing students' experiences of developing and demonstrating empathy while caring for patients with cancer. By gaining deeper insights into their perspectives and learning processes, this research contributes to a more comprehensive understanding of empathy in nursing education. The findings may also inform strategies for enhancing empathy training in clinical education, particularly for male nursing students.

## 2 Research Design

This qualitative study aimed to explore the experiences of Chinese male nursing students in developing and demonstrating empathy while caring for patients in oncology wards. Guided by the research question of how male nursing students experience the process of empathy development and expression in oncology care, the study sought to gain an in‐depth understanding of students' perceptions, learning processes and clinical interactions. A qualitative research design was considered most appropriate, as empathy is a complex, relational and context‐dependent phenomenon that is closely embedded in students' clinical learning and professional socialisation and is best explored through their subjective experiences rather than through quantitative measurement (Moorley and Cathala 2019). Moreover, qualitative research offers particular value in nursing education by illuminating students' learning experiences and the ways in which professional competencies are developed and enacted within real‐world clinical contexts (Moorley and Cathala 2019). This approach enabled a rich and nuanced exploration of how empathy is developed and demonstrated by male nursing students within the specific clinical and cultural context of oncology nursing practice in China.

This study employed a qualitative descriptive design, which is frequently used in nursing and healthcare research as it provides broad insight into specific phenomena and can be applied flexibly across a range of research contexts (Doyle et al. 2020). This approach is particularly useful for producing straightforward descriptions of experiences and perceptions, especially in areas where limited prior research exists (Kim et al. 2017). In the present study, the qualitative descriptive design enabled a clear and comprehensive description of Chinese male nursing students' experiences of developing and demonstrating empathy in oncology wards, while remaining close to participants' own accounts.

Although the study did not adopt a prescriptive theoretical model, it was informed by a conceptual framework drawing on literature related to empathy in nursing education and professional socialisation. Within this framework, empathy was conceptualised as a relational and developmental process shaped through students' clinical learning experiences and interactions with patients, particularly within the emotionally demanding context of oncology care. The framework also acknowledged the influence of students' positioning as male nursing students within a predominantly female profession. Consistent with the qualitative descriptive approach, the conceptual framework provided guidance for the development of the interview guide and functioned as a set of sensitising concepts during data analysis, without imposing predetermined theoretical assumptions or analytic categories. This allowed findings to emerge inductively from participants' narratives while supporting a systematic and transparent analytic process.

### 2.1 Sampling and Recruitment

This study utilised purposive sampling, which is conceptually equivalent to judgement sampling in qualitative research. This systematic approach enhances sample credibility through the intentional selection of information‐rich participants with in‐depth experience or specialised knowledge of the research topic (Shorten and Moorley 2014). Participant recruitment was conducted at the First Affiliated Hospital of Wannan Medical College in Anhui Province, China. The First Affiliated Hospital of Wannan Medical College comprises six cancer‐related wards, including general oncology wards, radiotherapy wards and haematology–oncology wards. Following approval from the university's Institutional Review Board (IRB), male nursing students were recruited if they met the following inclusion criteria: (1) completion of theoretical coursework and progression to the later stage of clinical practice; (2) current assignment to a clinical placement at the First Affiliated Hospital of Wannan Medical College; (3) a minimum of 1 month of clinical experience in an oncology ward; and (4) willingness to participate in the study. Nurse managers at the First Affiliated Hospital of Wannan Medical College were provided with these inclusion criteria and were asked to identify eligible students based solely on these criteria, without consideration of academic performance, clinical evaluations or perceived interpersonal qualities. Based on this process, a list of 26 eligible students was compiled, and all were invited to participate. Three students declined participation due to time constraints or personal reasons; therefore, a total of 23 male nursing students were included in the final sample.

Data saturation is widely recognised as a methodological principle in qualitative research (Saunders et al. 2018). In this qualitative descriptive study, code and thematic saturation was assessed concurrently with data collection and analysis. After analysis of the 21st interview, no new codes or themes emerged, and the data reflected repetition of previously identified patterns. The coding framework had stabilised, with subsequent interview data fitting within the existing codebook. To confirm saturation, two additional interviews were conducted. These interviews did not generate any new codes or themes relevant to the research aim, further supporting the judgement that saturation had been achieved. Consistent with the guidance of Fusch and Ness (2015), data collection was discontinued once sufficient depth and redundancy in the data were observed.

### 2.2 Data Collection

All participants provided informed consent before being interviewed. In‐depth and semi‐structured interviews were conducted by the fifth author (corresponding author), a senior male nurse with a doctoral degree in nursing and formal training in qualitative research. His professional background provided familiarity with the clinical context and facilitated rapport with participants; however, this positioning also carried the potential to influence data collection due to shared professional identity and perceived hierarchical relationships. The interviewer had no direct supervisory, teaching or evaluative relationship with the participating students and remained reflexively aware of his positioning throughout the interview process.

During the interviews, several strategies were employed to minimise potential power imbalances. Participants were informed that participation was voluntary, that there were no right or wrong answers, and that their responses would not affect their academic evaluation or clinical placement. Interviews were conducted in a private setting, and participants were encouraged to speak freely about both positive and challenging experiences. These strategies were intended to support open dialogue and enhance the credibility of the data.

Each participant was interviewed once in the conference room of the First Affiliated Hospital of Wannan Medical College, and each interview lasted about 30–60 min. Besides some demographic information on the participants, the questions in the interviews were as follows: (1) How would you define empathy in your own words? (2) In the cancer wards, how did you typically support patients or their families when you noticed they had concerns or needs—either through words or actions? (3) Can you describe a cancer patient who left a strong impression on you? What were your interactions with them like? (4) Did you find yourself showing empathy toward patients or their families? Can you share a specific situation and how you felt about it? and (5) During your clinical placements, were there any resources or experiences that helped you learn how to show empathy more effectively? The interview questions were developed through a review of relevant literature and were reviewed by experts in nursing education and qualitative research to enhance the credibility of the interview guide. During the interviews, the researcher would encourage the participants to openly share their experiences, thoughts and opinions. If any responses were unclear, the researcher would ask for clarification to ensure a better understanding.

### 2.3 Data Analysis

All 23 interviews were transcribed verbatim by the fifth author and analysed using thematic analysis following the framework proposed by Braun and Clarke (2006). The analytic process involved several iterative phases, including familiarisation with the data, generation of initial codes, searching for themes, reviewing and refining themes, and defining and naming the final themes.

During the familiarisation phase, all members of the research team read and reread the transcripts to gain an overall understanding of the data and to become immersed in participants' experiences of developing and demonstrating empathy in oncology wards. Initial coding was conducted by the fifth author, who systematically coded meaningful segments of text. Codes were data‐driven and assigned to segments that captured key ideas relevant to the research aim. To enhance analytic rigour, preliminary codes and emerging themes were discussed among the research team. Similarities and differences between codes were compared, and discrepancies in interpretation were resolved through discussion and consensus. Codes were then collated into potential subthemes and overarching themes by examining conceptual relationships and patterns across the dataset. NVivo (version 14) was used to organise and manage the data during the coding and analysis process. Through iterative comparison and refinement, three overarching themes and seven subthemes were identified that captured shared patterns across participants' accounts. An example of the coding process is presented in Table 1.

**TABLE 1:** An example of codes, subthemes and theme from content analysis of narratives about theme one.

Excerpts	Codes	Subthemes	Theme one
In school and when I first started clinical placement, I think the teachers and staff might have mentioned empathy, but they just briefly mentioned it.	Limited empathy education	Superficial understanding of empathy	Challenges in translating empathy into practice
I mostly learned about it from my phone, especially through social media.	Informal empathy learning		
I kind of understand that empathy means seeing things from the patient's perspective, but I don't really get the full meaning, and I'm not sure how to actually do it.	Uncertain application of empathy		
I feel that when working in a cancer ward, we should focus more on humanistic care and showing empathy, but I don't think I'm doing very well because I feel there's not much I can actually do.	Limited confidence in expressing empathy	Lack of confidence in expressing empathy	
For example, when I was in the Department of Oncology, I saw how weak those patients with cancer looked after chemotherapy. I felt really distressed and wanted to do something to help them, but I didn't know how, and I felt powerless.	Feelings of powerlessness in clinical situations		
One way is to carefully observe what senior nurses do, because they have a lot of clinical experience. By paying attention to the small details in their actions, you can really see their empathy.	Observing	Learning by observing, asking and experiencing	
For me, I usually ask our teachers directly what kinds of experiences are worth learning from. They've been through so much, and listening to their stories helps me understand how patients feel and how to respond to them more empathetically.	Actively asking		
From interacting with patients, I learned that it's important to respect them, try to put myself in their shoes, and show more care. Actually, very simple and ordinary actions can already show that you really value their feelings.	Learning from experience		

### 2.4 Translation and Verification

All interviews were conducted in Chinese and transcribed verbatim. The transcripts were translated into English by the third and fourth authors, who are fluent in both Chinese and English and have received formal training in qualitative research and academic writing. To ensure accuracy, the translation process focused on preserving the original meaning rather than producing a literal word‐for‐word translation. Cultural and contextual nuances were carefully considered throughout the translation process. In addition, the fourth author, who is also fluent in both languages and has extensive experience in qualitative research, cross‐checked selected English quotations against the original Chinese transcripts to ensure semantic consistency and clarity. Any discrepancies were discussed within the research team and resolved through consensus. To enhance transparency and confirmability, the original Chinese data were retained throughout the entire analytic process. Although a formal forward–back translation procedure was not employed, translation accuracy was ensured through iterative comparison between the Chinese transcripts and English translations and through ongoing discussion among members of the research team.

### 2.5 Rigour

In this study, trustworthiness was ensured by adhering to the four criteria proposed by Lincoln and Guba (1985): credibility, transferability, dependability and confirmability. The credibility of the findings was supported by all authors having experience in teaching and supporting male nursing students, enabling a deeper understanding of participants' experiences, challenges and potential issues. All participants in this study took part voluntarily and were open to sharing their personal experiences. During the interviews, the interviewer guided each participant in providing detailed accounts of their experiences, ensuring clarity and accuracy through follow‐up questions as needed. The analysis and reporting of participants' experiences were carefully documented. Lately, two participants were invited to review the key findings as part of the member‐checking process and confirmed that the findings aligned with their experiences, supporting the credibility of the study (Moorley and Cathala 2019). To enhance transferability, we included participants with diverse characteristics, such as variations in age, grades, educational institutions (universities and vocational schools) and clinical wards, and provided a detailed description of the study context, allowing readers to assess the applicability of the findings to other settings. Dependability was strengthened by having all interviews conducted by the fifth author, while the research team engaged in frequent discussions to refine data analysis, verify coding consistency and systematically organise themes. Lastly, confirmability was strengthened through the use of rich, descriptive excerpts that grounded the findings in participants' narratives. An audit trail was maintained throughout the research process to document methodological decisions, coding procedures and theme development, thereby enhancing transparency. In addition, the retention of the original Chinese transcripts and the cross‐checking of English translations further supported confirmability.

## 3 Findings

A total of 23 male nursing students participated in this study. Among them, 12 (52.2%) were enrolled in universities, while 11 (47.8%) attended vocational schools. Participants ranged in age from 19 to 23 years, with a mean age of 21.1 years. The total duration of clinical placement varied between 5 and 47 weeks, with an average of 14.1 weeks. Specifically, their oncology ward training periods ranged from 4 to 9 weeks, with a mean duration of 5.1 weeks. The details of the participants' socio‐demographic information are listed in Table 2.

**TABLE 2:** Participant characteristics (*N* = 23).

	Numbers	Percentages
Age (year)	Range = 19–23	Mean = 21.1
19	1	4.3%
20	4	17.4%
21	12	52.2%
22	4	17.4%
23	2	8.7%
Education		
University	12	52.2%
Vocational school	11	47.8%
Clinical placement experience (Week)	Range = 5–47	Mean = 14.1
04–08	9	39.1%
09–16	8	34.8%
17–24	2	8.7%
≥ 25	4	17.4%
Experience of oncology training (Week)	Range = 4–9	Mean = 5.1
9	1	4.3%
8	2	8.7%
7	1	4.3%
6	3	13.1%
5	3	13.1%
4	13	56.5%

We identified three themes and seven sub‐themes which captured the participants' clinical practice experiences of developing and demonstrating empathy when caring for patients in the oncology wards through learning by doing, as listed in Table 3.

**TABLE 3:** Themes and subthemes.

Themes	Sub‐themes
Theme 1: Challenges in Translating Empathy into Practice	1‐1 Superficial understanding of empathy 1‐2 Lack of confidence in expressing empathy 1‐3 Learning by observing, asking and experiencing
Theme 2: Determinants of Empathy	2‐1 Emotional resonance
	2‐2 Behaviour and attitudes of patients
Theme 3: Approaches to Demonstrating Empathy	3‐1 Difficulty in articulating empathy
	3‐2 Offering more practical support

### 3.1 Theme 1: Challenges in Translating Empathy Into Practice

#### 3.1.1 Subtheme 1‐1: Superficial Understanding of Empathy

All the male nursing student participants generally agreed on the importance of demonstrating empathy when caring for patients, particularly in cancer wards. However, their experiences indicated that formal education on empathy was not explicitly provided either in class or during clinical placements, was not emphasised enough to leave a lasting impression, or was not clearly remembered. Some participants were even uncertain whether it had been taught at all. Their understanding of empathy and related knowledge stemmed from various sources, including limited classroom instruction, social media, short videos, books and movies.

In school and when I first started clinical placement, I think the teachers and staff might have mentioned empathy, but they just briefly mentioned it. (Participant 1)

I don't remember it being taught in class‐I pretty much learned about it from my smart phone and social media. (Participant 5)

The reliance on varied and primarily informal sources for understanding empathy may account for participants' generally limited grasp of the concept. While some were able to provide a roughly accurate literal definition, their abilities to apply empathy meaningfully in patient care were inconsistent. Although some acknowledged its importance, they faced challenges in translating it into practice. Notably, many male nursing students struggled to clearly define empathy or articulate its application in clinical settings.

I think empathy means feeling the same way as others, understanding their ways, and recognizing their feelings. I think we should probably consider things from the other person's perspective, put ourselves in their shoes, think about the other person, and consider the issue from their perspective. (Participant 7)

I kind of understand that empathy means seeing things from the patient's perspective, but I don't really get the full meaning, and I'm not sure how to actually do it. (Participant 12)

#### 3.1.2 Subtheme 1‐2: Lack of Confidence in Expressing Empathy

Although most participants understood the basic concept of empathy, they generally felt that their knowledge, particularly their ability to express it, was still quite limited. They believed they could do little for their patients. As a result, even when they recognised a patient's distress during clinical placement, they lacked the confidence to demonstrate empathy and often felt uncertain or powerless.

I feel that when practice in the cancer ward, I should focus more on humanistic care and empathy, but I am not doing well because I feel that I can do very little. I basically just encourage and support them, with only simple verbal encouragement. I really want to do more, but I don't know how to do it. (Participant 18)

For example, when I was in the Department of Oncology, I saw how weak those patients with cancer looked after chemotherapy. I felt really distressed and wanted to do something to help them, but I didn't know how, and I felt powerless. (Participant 6)

#### 3.1.3 Subtheme 1‐3: Learning by Observing, Asking and Experiencing

Learning empathy is an ongoing process. Recognising their limitations in demonstrating empathy, some students actively sought strategies to enhance their skills. The majority acquired an understanding of how to express empathy through verbal and non‐verbal communication by observing interactions between senior clinical nurses or their mentors and patients.

Watching how senior nurses interact with patients taught me that it's important to respect, empathize with, and genuinely care for them. Honestly, even small things can make a big difference in showing patients you value their feelings. When I take care of patients, I try to call them by name in a warm, friendly way. I also make effort to talk to them while doing procedures, explain what's happening, and let them know what to expect. When giving injections, I reassure them and remind them the pain will pass quickly—it helps them feel more at ease. (Participant 20)

A small number of participants who were more motivated to learn would also acquire empathy‐related knowledge and skills through direct questioning.

I also consulted senior nurses directly to see what kind of experience is worth learning from. (Participant 11)

At some points, participants felt that the patients were also thinking about them from the students' perspectives. The participants were able to deeply experience the feeling of the patients empathising with them. This experience made them feel very good and indirectly reinforced their belief in the importance of empathy.

One time, I couldn't get a patient's injection right, so I immediately apologized. Patients with cancer often have fragile veins, and too many attempts can cause damage. But instead of getting upset, the patient reassured me, saying it was okay. He even comforted me, saying that every young nurse goes through this and that even senior nurses started the same way, learning step by step. He told me not to feel too much stress and to take my time. His encouragement made me feel understood, and it really touched me. (Participant 19)

### 3.2 Theme 2: Determinants of Empathy

For participants, the demonstration of empathy was shaped by two key factors: the presence of emotional resonance in the care situation and the patient's behaviour and attitude during the care process.

#### 3.2.1 Subtheme 2‐1: Emotional Resonance

Most participants observed that patients in cancer wards experience multiple forms of suffering. The long and challenging treatment process places a significant burden not only on their physical health but also on their families and financial situations. Given these hardships, participants recognised that it was natural for patients to have various emotional responses, and they gained a deeper understanding of the emotional struggle patients face throughout their treatment journey.

They had been receiving treatment for so long, but their condition had not improved, so they were a little frustrated. Also, they had spent so much money, they were under a lot of financial pressure, and sometimes they got angry. There was a patient who was very optimistic when he was first admitted to the hospital. But after three weeks of hospitalization, he lost his temper with his family members and was sometimes dissatisfied with us medical workers. This is very common and we can understand it. (Participant 22)

Additionally, when certain patient characteristics—such as young age—or specific situations evoked personal experiences among the participants during the care process, they tended to develop a stronger emotional resonance. This connection often enhanced their motivation to express greater empathy and provide additional support to the patient.

When we saw them, we couldn't help but wonder‐ why would such young kids have to suffer from tumors? That little boy with rectal cancer was so thin, barely any meat on his bones. And yet, he was still holding on, going through chemotherapy just to stay alive. It was really heartbreaking to watch. Kids their age should be carefree, focusing on school and enjoying life. But instead, they're battling illness, going through surgeries and chemotherapy. It's really painful to see. (Participant 6)

Because my grandfather also had surgery in the hospital for esophageal cancer, I used to take care of my grandfather often, and I felt in my heart that he was a bit similar to my grandfather, so I had a special feeling for him. (Participant 17)

#### 3.2.2 Subtheme 2‐2. Behaviour and Attitudes of Patients

According to the participants' experiences, when patients had a positive attitude toward treatment, were willing to accept treatment and restore their personal health, and showed kindness toward students' care, the participants tended to be more willing to interact with the patients and had a greater willingness to show empathy.

I think his behavior was quite different from that of most patients with cancer. Every time I treated him, he had a smile on his face. He was incredibly positive and optimistic, always treating others with kindness and respect, which made us respect him even more in return. A smile is contagious. Whenever I took care of him, his smile lifted my mood as well. I also felt that he received a bit of special care. I was extra cautious and gentle with him, making sure every procedure was done with the utmost care. (Participant 13)

Conversely, patients' resistance to treatment, expressions of anger or irritability or even unreasonable outbursts toward the participants could diminish their motivation to demonstrate empathy. When faced with rejection or aggressive behaviour, the participants might experience anxiety or emotional strain. To shield themselves from the impact of these negative emotions during patient care, they might unintentionally create emotional distance, leading to a reduction in their empathetic engagement. Practically, in such situations, the participants often prioritised self‐preservation by maintaining a distance from the patient, limiting their interactions and providing only essential care without much verbal or emotional engagement.

There were a lot of angry patients in the cancer ward. There was a patient who I went to give an interleukin injection to. Right after the injection, he asked me why I was in such a hurry to give him the injection, and then came over and scolded us … I thought I was doing this for the patient's good, but he not only didn't appreciate it, he blamed us instead. So when I was treating this patient, I became very upset. I felt that he shouldn't be like this and shouldn't disrespect us. (Participant 8)

I felt like that patient was ungrateful, so after a while, I didn't really want to give him too much attention, talk to him much, or spend my energy on him. That's how I felt at the time, though I knew it wasn't the right mindset. Still, I kept my distance. But if he needed help, I would help him—because, at the end of the day, that's just part of the job. I couldn't ignore that. (Participant 4)

### 3.3 Theme 3: Approaches to Demonstrating Empathy

The participants' experiences indicated that, while most understood that empathy should ideally be expressed through both verbal and non‐verbal actions, they found it more challenging to convey empathy through words. In contrast, they felt more comfortable demonstrating empathy through direct, supportive actions that met patients' needs.

#### 3.3.1 Subtheme 3‐1: Difficulty in Articulating Empathy

Most of the participants found themselves unable to provide emotional comfort to soothe or calm down clients when they needed verbal comfort.

Relatively speaking, I am not very good at counseling patients. I just chat with them occasionally, observe them briefly, and ask them how they have been doing recently, but there is no in‐depth communication. It's just a normal chat. (Participant 9)

Some participants believed that gender characteristics also affected the individual's expression of concern for patients or comfort for patients. They believed that, compared with women, men were naturally less able to express empathy, but some participants did not agree with this view.

Women tend to express empathy more easily—I feel like they're better at comforting patients. (Participant 21)

But in the end, it really depends on the individual. While it's true that women are generally more detail‐oriented than men, it's not always the case. Some men can be very sensitive and thoughtful, while some women can be more straightforward and less emotionally expressive. It all comes down to personality—it varies from person to person. (Participant 16)

#### 3.3.2 Subtheme 3‐2: Offering More Practical Support

Most participants expressed empathy primarily through their actions. They believed that genuinely understanding a patient's physical condition and health needs, as well as providing care with heightened caution and gentleness to prevent further discomfort or harm, was a meaningful demonstration of empathy.

For such a patient, you will sincerely pity and sympathize with him, and then I will be more gentle when I encounter this kind of disease during the subsequent operation, and use both hands as much as possible. Sometimes using one hand may cause a pull on the arm, which may lead to this kind of fracture. (Participant 5)

Mainly, I will be very careful in the operation and will tell him what I am going to do first, and then hope that he can cooperate with me in the operation. Through his cooperation, I can reduce the pulling during the nursing process and avoid causing him any harm. When caring for the patient, we will be very careful, reduce pulling as much as possible, and perform nursing operations slowly and carefully. (Participant 7)

Sometimes, the participants were limited by their own technical abilities and thus unable to do a good job. To protect patients and avoid unnecessary pain and harm, they sought timely help from doctors or senior nurses.

Such a young child is undergoing chemotherapy, so his blood vessel condition is not very good. Our ability is not good either, so we'd better let the teacher do the injection. After all, the teacher's technique is better and he won't suffer more. (Participant 10)

## 4 Discussion

This study used a qualitative research method to interview 23 male nurses from different schools and learned from their experiences how they developed, demonstrated and expressed empathy in the process of caring for patients with cancer in cancer wards. We identified three themes: (1) challenges in translating empathy into practice, (2) determinants of empathy and (3) approaches to demonstrating empathy. There are some key issues that deserve further discussion.

### 4.1 Challenges in Translating Empathy Into Practice

The results of this study showed that the participants recognised the importance of empathy in clinical patient care, especially in the cancer ward. Empathy skills are particularly important in the oncology setting, as patients and their families often experience significant psychological distress, including depression (Lyu et al. 2024). It has been argued that early exposure to empathy training can better prepare nursing students for real‐world clinical practice, reducing the risk of entering the workforce without adequate skills to support emotionally distressed patients (Huang et al. 2025). Unfortunately, from the participants' experiences, it seems that these male nursing students had not learned the relevant theories and concepts of empathy in formal education either in school or in clinical placement. Although students can obtain empathy‐related content from various informal sources (such as social media), there are still questions about how accurate and complete these materials are and whether they are sufficient for male nursing students to apply in the clinical care of patients. Even when students have some understanding of empathy, they often lack confidence in their ability to express it appropriately in clinical care. Many perceive themselves as lacking the necessary skills, leading to feelings of uncertainty and even frustration. In fact, research has shown that male and female nursing students demonstrate comparable levels of empathy (Deng et al. 2023). Thus, male students' lack of confidence may not reflect their actual ability but rather the influence of traditional gender stereotypes, which can undermine their confidence in expressing empathy. By placing greater emphasis on empathy development through targeted training programs, nursing educators can help challenge these stereotypes and enable male nursing students to provide patients with the same level of compassion and care as their female counterparts (Deng et al. 2023). Additionally, our study also found that students develop their empathy‐related knowledge and skills through various approaches in clinical practice. For example, they learn by observing how experienced nurses demonstrate empathy while caring for patients. These are undoubtedly valuable learning strategies and should be encouraged. However, we recommend that nursing educators incorporate structured courses or instructional activities both in academic settings and during clinical training (Huang et al. 2025). Providing students with a deeper understanding of empathy and practical training in empathy‐related skills would ensure more effective learning and better application in clinical practice (Ozcan et al. 2012). In particular, by tailoring teaching strategies to the learning characteristics of male nursing students, empathy can be more effectively cultivated. Empathy has been described as a cognitive skill that can be invoked, taught, re‐awakened and nurtured. In clinical settings, this implies that training should emphasise strengthening active listening through practice and reflection. Equally important is the role of mentoring, as teachers who consistently serve as role models of reflective listening provide students with opportunities to observe, learn and be inspired to adopt similar behaviours (Thangarasu et al. 2021). Building on this, structured mentorship or role‐modelling programs could serve as intentional strategies for fostering empathy, particularly among male students who may encounter cultural or gender‐related barriers to emotional expression. Such formalised mentorship offers safe and guided opportunities for reflective practice and emotional engagement, thereby enhancing the practical applicability of the study's findings.

### 4.2 Determinants of Empathy

This study identified two key factors, beyond existing knowledge and skills, that influence students' expression of empathy in clinical care, either facilitating or inhibiting it. In oncology wards, the male nursing students in the current study demonstrated a natural tendency to assist patients experiencing greater physical, psychological or financial distress, a result that aligns with previous findings (Lyu et al. 2024). This behaviour suggests that nursing empathy is not solely an individual disposition or personal commitment but rather a collaboratively constructed process, shaped by specific interactional contexts (Wu 2021). In other words, empathy in nursing does not arise in isolation from the caregiver's intrinsic qualities; instead, it develops dynamically through engagement with patients, influenced by real‐time interactions, observed suffering and the clinical environment. However, a more compelling observation was that the students demonstrated heightened emotional engagement and a stronger motivation to help when caring for patients who shared similar characteristics, such as age, or whose experiences resonated with their own personal histories (Bonacaro et al. 2024). This perspective highlights the situational and relational nature of nursing empathy, emphasising that it emerges as a response to external stimuli rather than existing as a static personal trait.

For the male nursing students in the current study, the attitudes and behaviours of patients significantly influenced their willingness to express empathy. When they encountered patients who had a positive and proactive attitude toward treatment or who were friendly toward them, they were more inclined to demonstrate empathy at the appropriate moments. Conversely, when faced with difficult patients, such as those who became angry or irritable for no apparent reason, these students tended to distance themselves as a form of self‐preservation. Interestingly, other studies have shown that oncology patients value attitudes, behaviours and personal traits of specific clinical professionals that can improve their care experience (Sanders et al. 2021). Therefore, training nursing staff in high‐quality communication skills, such as active listening and a holistic patient focus, can enhance empathy in clinical practice (Sanders et al. 2021). This phenomenon especially highlights a critical learning gap: While it is natural to withdraw from challenging interactions, nursing students also need to develop skills to navigate such situations effectively. Rather than avoiding patients who appear angry or uncooperative, they should be encouraged to explore the underlying reasons for their emotions and respond with appropriate empathy. This suggests an important area for nursing education. Educators could design specialised lesson plans to help male nursing students better understand and manage interactions with emotionally distressed or difficult patients. Providing structured learning experiences on how to maintain empathy in challenging clinical encounters could better prepare them for real‐world patient care (Huang et al. 2025).

### 4.3 Approaches to Demonstrating Empathy

In contrast, some studies suggest that male nurses generally exhibit lower empathy skills and tendencies compared to their female counterparts (Alkan 2017). However, the participants in our study expressed diverse perspectives, with findings aligning with the argument that the perceived superiority of women's empathy remains ambiguous—whether it arises from biological or social differences between genders or merely reflects prevailing gender role stereotypes (Pang et al. 2023). Alternatively, male and female caregivers may simply express empathy in distinct ways (Cunico et al. 2012). Additionally, our research offers a deeper understanding of this phenomenon. Although the male nursing students acknowledged the importance of expressing empathy toward certain patients, most struggled to provide clear, meaningful and supportive verbal responses. Studies indicate that fostering hope, employing distraction techniques, incorporating spirituality and assisting patients in adapting to new circumstances can help cultivate an environment that enhances meaningful empathic interactions (Babaii et al. 2021). However, despite their attempts to provide verbal comfort, the male nursing students in this study often felt constrained to casual conversations and simple words of reassurance or encouragement, which they perceived as superficial and insufficient to convey genuine empathy. They also regarded themselves as less adept at expressing and demonstrating empathy than their female counterparts, even though this may not necessarily be the case (Deng et al. 2023). Consistent with Connell's concept of hegemonic masculinity, such perceptions may reinforce a tendency among male nurses to suppress personal emotions and expressions of care, fearing that these behaviours might be interpreted as feminine or unmanly (Connell and Messerschmidt 2005).

Additionally, influenced by traditional Confucian culture, core values such as ‘*Ren*’ (humaneness) emphasise compassion, empathy and kindness toward others (Nie and Jones 2019), while ‘*Xiao*’ (filial piety) highlights respect and care for parents and elders, extending naturally to respect for patients (Wesołowski 2022). These values closely align with the nursing ideals of empathy, caring and patient‐centeredness. Thus, even though male nursing students may face questions about not conforming to conventional notions of masculinity when entering the nursing profession, the Confucian ethics of ‘*Ren*’ and ‘*Xiao*’ provide cultural legitimacy for their choice. Therefore, the male nursing students in this study did not entirely reject the expression of empathy; rather, they preferred to convey it in ways that felt more natural and comfortable to them. An interesting phenomenon observed was that, in clinical practice, male nursing students were more inclined to express empathy and foster patients' confidence through actions rather than words. They often assisted patients with tasks, provided additional physical support, and demonstrated gentleness in their care. This preference for action‐based empathy appeared to stem from a lack of confidence in verbal communication. Many participants feared saying the wrong thing, struggled to articulate empathy effectively or worried that their attempts at expressing empathy might upset patients.

Importantly, the findings suggest that action‐based expressions of empathy should not be interpreted as a deficit or a substitute for verbal empathy. Rather, within the Chinese cultural and clinical context, such expressions represent a legitimate and culturally congruent mode of empathic care. Drawing on Confucian values that emphasise responsibility, benevolence (Ren), and practical care for others, male nursing students often expressed empathy through attentive presence, practical assistance and consistent support. These behaviours reflect an embodied and relational form of empathy aligned with cultural expectations and professional norms, and should be recognised as a valid expression of empathic nursing practice.

In this sense, the male nursing students in this study can be understood as implicitly redefining empathy. Instead of adhering to a Western conceptualisation that prioritises verbal emotional mirroring and explicit emotional disclosure, their accounts point to an understanding of empathy grounded in practical assistance, task competence and respectful service. Empathy was enacted through reliably meeting patients' needs, providing physical support and demonstrating attentiveness through action rather than emotionally expressive language. This redefinition does not signal a rejection of empathy, but a reframing of how empathy is understood and enacted within a specific cultural, educational and professional context. Recognising this shift broadens prevailing definitions of empathy in nursing and challenges assumptions that verbal emotional expression constitutes the primary or most authentic indicator of empathic care.

Beyond gendered expectations, the findings can also be understood in relation to the cultural significance of ‘mianzi’ (face) and the value placed on emotional restraint within Chinese society. In Confucian‐influenced contexts, maintaining face involves regulating emotional expression to avoid embarrassment, discomfort or perceived impropriety for oneself and for others. For male nursing students, verbally articulating deep emotional empathy may carry the risk of saying the ‘wrong’ thing, appearing overly emotional or inadvertently causing patients to lose ‘mianzi’.

Within this cultural framework, action‐based expressions of empathy may represent a safer and more socially acceptable approach to care. By offering practical assistance, attentive presence and respectful service, students are able to demonstrate concern and support without violating cultural norms surrounding emotional reserve. Thus, difficulties with verbal empathy expression should not be interpreted solely as a skill deficit, but as a culturally situated response shaped by the interplay of face concerns, professional expectations and educational context.

Beyond cultural influences, the findings may also be shaped by professional socialisation processes and curriculum structures in nursing education. Clinical training often prioritises technical competence, task completion and efficiency, while opportunities to practice and receive feedback on verbal emotional communication may be limited. Within such educational contexts, male nursing students may learn to demonstrate competence and care primarily through action‐oriented behaviours, reinforcing action‐based empathy as both professionally appropriate and practically valued. These educational and professional norms interact with cultural expectations to shape how empathy is learned and enacted in clinical practice.

Taken together, these findings highlight the need for educational strategies that explicitly support diverse expressions of empathy while strengthening students' confidence in verbal emotional communication. Empathy‐focused simulations, such as objective structured clinical examinations (OSCEs) incorporating emotionally complex or challenging patient scenarios, reflective journaling, role‐play exercises and supervised debriefing sessions, have been shown to enhance empathic understanding and communication in nursing education (Jiao et al. 2022; Peisachovich et al. 2024). Such approaches may be particularly valuable for male nursing students by validating action‐based empathy as a legitimate form of care, while also providing structured opportunities to develop verbal empathic skills (Levett‐Jones et al. 2019). Strengthening these competencies may foster stronger nurse–patient relationships, improve the quality of patient care and ultimately enhance overall healthcare outcomes.

## 5 Limitation

This study has several limitations that should be considered when interpreting the findings. First, the study recruited 23 male nursing students from a single hospital in Anhui Province using purposive sampling. The limited sample size and focus on one geographical region may not fully capture the diverse experiences of male nursing students across China. In addition, recruitment through nurse managers may have introduced selection bias, as managers could have preferentially recommended students perceived as more engaged or compliant. Although all eligible students were informed that participation was voluntary and that confidentiality and anonymity would be maintained, this recruitment approach may nevertheless have influenced the range and authenticity of perspectives represented in the data.

Second, interviewer–participant dynamics may have influenced the interview process. The interviews were conducted by a senior male nurse, whose professional background may have facilitated rapport and contextual understanding. However, the interviewer's seniority and professional status may also have led participants to moderate their responses, align with perceived professional expectations or withhold more vulnerable perspectives, particularly when discussing emotionally sensitive topics such as empathy.

Third, as interviews were conducted in Chinese and subsequently translated into English, there remains a risk that subtle semantic meanings, emotional tones or culturally embedded nuances were not fully preserved, despite rigorous translation and verification procedures. This represents an inherent limitation of cross‐linguistic qualitative research.

Fourth, given the sensitivity of the topic, social desirability bias may have occurred, with participants shaping their responses to align with socially and professionally valued norms of caring and empathy. Finally, the study relied solely on semi‐structured interviews for data collection. Future research could strengthen the richness and trustworthiness of the findings by incorporating additional data sources, such as observational data, reflective journals or clinical field notes.

## 6 Conclusion

This study indicates that male nursing students receive limited structured education on demonstrating empathy, both in the classroom and during clinical placements. Although they may develop a basic understanding of empathy, translating this understanding into practice, particularly through verbal communication, remains challenging. Their willingness to express empathy is also shaped by their interactions with patients. To address these challenges, nursing educators should consider the unique characteristics of male nursing students, including their learning styles and their tendency to express empathy through actions rather than words. Well‐designed courses and collaborative learning activities integrated across academic and clinical settings may enhance empathy development and strengthen students' professional identities.

Further research is warranted to explore differences in the learning, development and expression of empathy between male and female nursing students. A deeper understanding of male students' preference for action‐based expressions of empathy may help refine educational strategies and ultimately improve patient care outcomes. Importantly, this study highlights action‐based empathy as a legitimate and culturally grounded form of empathic care among Chinese male nursing students, underscoring the need for nursing education to recognise and support diverse expressions of empathy.

## Author Contributions

Conception and design: Xue Chen, Xiao‐Chen Lyu and Cheng‐I Yang. Data collection and analysis: Xue Chen, Xiao‐Chen Lyu, Fang‐Tuan Wu and Li‐Hung Lee. Manuscript preparation: Xue Chen, Xiao‐Chen Lyu and Cheng‐I Yang.

## Funding

Funding received from the Scientific Research Project of Higher Education Institutions in Anhui Province (Grant Number: 2023AH053355).

## Ethics Statement

Ethical approval was received from the Ethics Committee of Wannan Medical College (Approval: No. WNMC‐2023‐203). This study was conducted in accordance with the Declaration of Helsinki. The authors confirm that all methods were performed in accordance with the relevant guidelines and regulations.

## Consent

Informed consent was obtained from all participants before the interview. The quotations used in this study were anonymised to ensure confidentiality.

## Conflicts of Interest

The authors declare no conflicts of interest.

## Data Availability

The data that support the findings of this study are available from the corresponding author upon reasonable request.

## References

[nop270481-bib-0001] Alkan, A. 2017. “The Effects of Nurses' Empathy Skills on Attitudes Towards Patients With Cancer.” Journal of Clinical and Experimental Investigations 8, no. 2: 61–65. 10.5799/jcei.333383.

[nop270481-bib-0002] Babaii, A. , E. Mohammadi , and A. Sadooghiasl . 2021. “The Meaning of the Empathetic Nurse–Patient Communication: A Qualitative Study.” Journal of Patient Experience 8: 1–9. 10.1177/23743735211056432. PMC8640307 34869836

[nop270481-bib-0003] Bonacaro, A. , F. Cortese , C. Taffurelli , A. Sollami , C. Merlini , and G. Artioli . 2024. “The Empathetic Involvement of Nurses in the Context of Neuroscience: A Mixed‐Methods Study.” Healthcare 12, no. 20: 2081. 10.3390/healthcare12202081. 39451495 PMC11507446

[nop270481-bib-0004] Braun, V. , and V. Clarke . 2006. “Using Thematic Analysis in Psychology.” Qualitative Research in Psychology 3, no. 2: 77–101. 10.1191/1478088706qp063oa.

[nop270481-bib-0005] Cassels, T. G. , S. Chan , and W. Chung . 2010. “The Role of Culture in Affective Empathy: Cultural and Bicultural Differences.” Journal of Cognition and Culture 10, no. 3–4: 309–326. 10.1163/156853710X531203.

[nop270481-bib-0006] Connell, R. W. , and J. W. Messerschmidt . 2005. “Hegemonic Masculinity: Rethinking the Concept.” Gender & Society 19, no. 6: 829–859.

[nop270481-bib-0007] Cunico, L. , R. Sartori , O. Marognolli , and A. M. Meneghini . 2012. “Developing Empathy in Nursing Students: A Cohort Longitudinal Study.” Journal of Clinical Nursing 21, no. 13–14: 2016–2025. 10.1111/j.1365-2702.2012.04105.x. 22672461

[nop270481-bib-0008] Deng, X. , S. Chen , X. Li , et al. 2023. “Gender Differences in Empathy, Emotional Intelligence and Problem‐Solving Ability Among Nursing Students: A Cross‐Sectional Study.” Nurse Education Today 120: 105649. 10.1016/j.nedt.2022.105649. 36435156

[nop270481-bib-0009] Doyle, L. , C. McCabe , B. Keogh , A. Brady , and M. McCann . 2020. “An Overview of the Qualitative Descriptive Design Within Nursing Research.” Journal of Research in Nursing 25, no. 5: 443–455. 10.1177/1744987119880234. 34394658 PMC7932381

[nop270481-bib-0010] Fusch, P. I. , and L. R. Ness . 2015. “Are We There Yet? Data Saturation in Qualitative Research.” Qualitative Report 20, no. 9: 1408–1416. 10.46743/2160-3715/2015.2281.

[nop270481-bib-0011] Heggestad, A. K. T. , P. Nortvedt , B. Christiansen , and A. S. Konow‐Lund . 2018. “Undergraduate Nursing Students' Ability to Empathize: A Qualitative Study.” Nursing Ethics 25, no. 6: 786–795. 10.1177/0969733016664982. 27605557

[nop270481-bib-0012] Huang, H. P. , Y. Tien , Y. C. Lin , I. C. Yu , and N. H. Chien . 2025. “Effects of Empathy Mapping and Mini‐Simulation on Second‐Year Nursing Students' Empathy and Communication Self‐Confidence: A Quasi‐Experimental Study.” BMC Medical Education 25: 109. 10.1186/s12909-025-06686-x. 39844127 PMC11755898

[nop270481-bib-0013] Jiao, J. R. , Y. X. Zheng , and W. N. Hao . 2022. “Empathy Ability of Nursing Students: A Systematic Review and Meta‐Analysis.” Medicine 101, no. 32: e30017. 10.1097/MD.0000000000030017. 35960092 PMC9371485

[nop270481-bib-0014] Kim, H. , J. S. Sefcik , and C. Bradway . 2017. “Characteristics of Qualitative Descriptive Studies: A Systematic Review.” Research in Nursing & Health 40, no. 1: 23–42. 10.1002/nur.21768. 27686751 PMC5225027

[nop270481-bib-0015] Levett‐Jones, T. , R. Cant , and S. Lapkin . 2019. “A Systematic Review of the Effectiveness of Empathy Education for Undergraduate Nursing Students.” Nurse Education Today 75: 80–94. 10.1016/j.nedt.2019.01.006. 30739841

[nop270481-bib-0016] Lincoln, Y. S. , and E. G. Guba . 1985. Naturalistic Inquiry. Sage.

[nop270481-bib-0017] Low, K. C. P. 2012. “Being Empathetic, the Way of Confucius.” Educational Research 3, no. 11: 818–826.

[nop270481-bib-0018] Lyu, X. C. , H. J. Jiang , L. H. Lee , C. I. Yang , and X. Y. Sun . 2024. “Oncology Nurses' Experiences of Providing Emotional Support for Cancer Patients: A Qualitative Study.” BMC Nursing 23, no. 1: 58. 10.1186/s12912-024-01718-1. 38245735 PMC10800062

[nop270481-bib-0019] Moorley, C. , and X. Cathala . 2019. “How to Appraise Qualitative Research.” Evidence‐Based Nursing 22, no. 1: 10–13. 10.1136/ebnurs-2018-103044. 30504448

[nop270481-bib-0020] Nie, J. B. , and D. G. Jones . 2019. “Confucianism and Organ Donation: Moral Duties From Xiao (Filial Piety) to Ren (Humaneness).” Medicine, Health Care and Philosophy 22, no. 4: 583–591. 10.1007/s11019-019-09893-8. 30903406

[nop270481-bib-0021] Ozcan, C. T. , F. Oflaz , and B. Bakir . 2012. “The Effect of a Structured Empathy Course on the Students of a Medical and a Nursing School.” International Nursing Review 59, no. 4: 532–538. 10.1111/j.1466-7657.2012.01019.x. 23134138

[nop270481-bib-0022] Pang, C. , W. Li , Y. Zhou , T. Gao , and S. Han . 2023. “Are Women More Empathetic Than Men? Questionnaire and EEG Estimations of Sex/Gender Differences in Empathic Ability.” Social Cognitive and Affective Neuroscience 18, no. 1: 1–16. 10.1093/scan/nsad008. PMC9976760 36807483

[nop270481-bib-0023] Peisachovich, E. H. , E. V. Sombilon , N. Graham , N. Ladha , and C. D. Silva . 2024. “Evaluating the Effectiveness of Empathy‐Based Education in Undergraduate Nursing: A Scoping Review.” Journal of Nursing Education 63, no. 6: 367–371. 10.3928/01484834-20240404-01. 38900258

[nop270481-bib-0024] Sanders, J. J. , M. Dubey , J. A. Hall , H. Z. Catzen , D. Blanch‐Hartigan , and R. Schwartz . 2021. “What Is Empathy? Oncology Patient Perspectives on Empathic Clinician Behaviors.” Cancer 127, no. 22: 4258–4265. 10.1002/cncr.33834. 34351620

[nop270481-bib-0025] Saunders, B. , J. Sim , T. Kingstone , et al. 2018. “Saturation in Qualitative Research: Exploring Its Conceptualization and Operationalization.” Quality & Quantity 52, no. 4: 1893–1907. 10.1007/s11135-017-0574-8. 29937585 PMC5993836

[nop270481-bib-0026] Shorten, A. , and C. Moorley . 2014. “Selecting the Sample.” Evidence‐Based Nursing 17, no. 2: 32–33. 10.1136/eb-2014-101747. 24561511

[nop270481-bib-0027] Thangarasu, S. , G. Renganathan , and P. Natarajan . 2021. “Empathy Can Be Taught, and Patients Teach It Best.” Journal of Medical Education and Curricular Development 8: 23821205211000346. 10.1177/23821205211000346. 33796792 PMC7975442

[nop270481-bib-0028] Wang, Q. , X. Cao , and T. Du . 2022. “First‐Year Nursing Students' Initial Contact With the Clinical Learning Environment: Impacts on Their Empathy Levels and Perceptions of Professional Identity.” BMC Nursing 21, no. 1: 234. 10.1186/s12912-022-01016-8. 35999595 PMC9400203

[nop270481-bib-0029] Wesołowski, Z. 2022. “The Virtues of Xiao (Filial Piety) and Ti (Brotherly Obedience) as Two Pillars of Confucian Familism.” Studia Warmińskie 59: 315–336. 10.31648/sw.8336.

[nop270481-bib-0030] Wu, Y. 2021. “Empathy in Nurse‐Patient Interaction: A Conversation Analysis.” BMC Nursing 20: 18. 10.1186/s12912-021-00535-0. 33435957 PMC7802140

